# Norepinephrine Induces Lung Microvascular Endothelial Cell Death by NADPH Oxidase-Dependent Activation of Caspase-3

**DOI:** 10.1155/2020/2563764

**Published:** 2020-02-12

**Authors:** Andreia Z. Chignalia, Guy Weinberg, Randal O. Dull

**Affiliations:** ^1^College of Medicine, Department of Anesthesiology, University of Illinois at Chicago, Chicago, Illinois, USA; ^2^College of Medicine, Department of Anesthesiology, University of Arizona, Tucson, Arizona, USA; ^3^College of Medicine, Department of Physiology, University of Arizona, Tucson, Arizona, USA

## Abstract

Norepinephrine (NE) is the naturally occurring adrenergic agonist that is released in response to hypotension, and it is routinely administered in clinical settings to treat moderate to severe hypotension that may occur during general anesthesia and shock states. Although NE has incontestable beneficial effects on blood pressure maintenance during hypotensive conditions, deleterious effects of NE on endothelial cell function may occur. In particular, the role of reactive oxygen species (ROS) and NADPH oxidase (Nox) on the deleterious effects of NE on endothelial cell function have not been fully elucidated. Therefore, we investigated the effects of NE on ROS production in rat lung microvascular endothelial cells (RLMEC) and its contribution to cell death. RLMEC were treated with NE (5 ng/mL) for 24 hours and ROS production was assessed by CellROX and DCFDA fluorescence. Nox activity was assessed by NADPH-stimulated ROS production in isolated membranes and phosphorylation of p47phox; cell death was assessed by flow cytometry and DNA fragmentation. Caspase activation was assessed by fluorescent microscopy. Nox1, Nox2, and Nox4 mRNA expression was assessed by real-time PCR. NE increased ROS production, Nox activity, p47phox phosphorylation, Nox2 and Nox4 mRNA content, caspase-3 activation, and RLMEC death. Phentolamine, an *α*_1_-adrenoreceptor antagonist, inhibited NE-induced ROS production and Nox activity and partly inhibited cell death while *β*-blockade had no effect. Apocynin and PEGSOD inhibited NE-induced caspase-3 activation and cell death while direct inhibition of caspase-3 abrogated NE-induced cell death. PEG-CAT inhibited NE-induced cell death but not caspase-3 activation. Collectively, these results indicate that NE induces RLMEC death via activation of Nox by *α*-adrenergic signaling and caspase-3-dependent pathways. NE has deleterious effects on RLMECs that may be important to its long-term therapeutic use.

## 1. Introduction

Norepinephrine (NE) is the endogenous catecholamine released by sympathetic nerve fibers in the vascular wall in response to acute reductions in blood pressure. Exogenous NE is commonly used to support blood pressure during shock due to its dual action on both *α*_1_- and *β*_1_-adrenoreceptors that, respectively, cause vasoconstriction and increase myocardial contractility, ultimately resulting in increased peripheral vascular resistance and cardiac output. However, studies in rats indicate that typical pressor doses of NE or epinephrine can alter pulmonary capillary integrity leading to accumulation of lung water and ultimately pulmonary edema [1, 2]. These reports reveal an important and yet unexplored area: the potential long-term side effects of the use of catecholamines in clinical settings. Whether NE exerts deleterious effects on vascular cells is still unknown and is worth of exploration as it may have direct bearing on the clinical use of catecholamines. In particular, understanding the potential deleterious effects of pathophysiological concentrations of NE in lung endothelial cells may be critical as it has been shown that these cells develop dysfunction, and, specifically barrier failure, when acutely exposed to NE. Moreover, the lung endothelium uptakes approximately 30% of the circulating NE, making this organ an important target for NE.

The effects of NE on endothelial function have been related to increased reactive oxygen species (ROS) production. We have recently shown that, in rats, continuous infusion of NE for two hours leads to nitric oxide synthase (NOS) uncoupling in the lungs. Uncoupled NOS then drives superoxide anion generation leading to oxidative stress, endothelial dysfunction, and endothelial barrier disruption that ultimately causes pulmonary edema [2]. Additionally, it has been shown that NE increases ROS production by a NADPH oxidase1 (Nox1) dependent pathway in human peripheral mononuclear cells [3] and in rat endothelial cardiac cells [4]. Interestingly, ROS production by NE can be mediated either by *α*- [3] or *β*-adrenoceptors depending on the cell type [5].

The suprapharmacological concentrations of NE used in many studies have limited clinical significance. We therefore investigated the effects of a clinically relevant concentration of NE [6–8] on Nox activity and ROS production in endothelial cells and its contribution to endothelial cell death. Herein, we report that NE, in a concentration in the same order of magnitude as the ones found in human plasma of critically ill patients, can be toxic to rat lung microvascular endothelial cells.

## 2. Methods

### 2.1. Drugs and Reagents

Chemicals were the highest grade available. Norepinephrine bitartrate salt, apocynin, propranolol, phentolamine, superoxide dismutase-polyethylene glycol (PEG-SOD), and catalase-polyethylene glycol (PEG-CAT) were purchased from Sigma Chemicals. Phospho-p47phox (Ser370) was purchased from Invitrogen. Caspase-3 inhibitor III, was purchased from Santa Cruz Biotechnologies (Dallas, TX). FITC Annexin V/Dead Cell Apoptosis Kit®, CellROX® probe, 5-(and-6)-carboxy-2′,7′-dichlorodihydrofluorescein, CellEventTM® kit, Alexa Fluor ™ 647 goat anti-rabbit antibody, and one step Power SYBR™ Green RNA to CT™ RT-PCR kit were purchased from Thermo Fisher Scientific (Waltham, MA). FragEL™ DNA Fragmentation Detection Kit Fluorescence TdT enzyme was purchased from EMD Millipore (Billerica, MA).

### 2.2. Cell Culture

Rat lung microvascular endothelial cells (RLMEC) were purchased from VEC Technologies (Rensselaer, NY) and cultured with EBM-2 medium (Lonza, Walkersville, MD) supplemented with 10% fetal bovine serum; cells were maintained in an incubator at 37°C, 5% CO_2_. Cells from passage 4 to 10 were used in these experiments. Experiments were performed using NE 5 ng/mL (15.7 nM). The concentration of NE was chosen based on a cell viability concentration-response curve, assessed by trypan blue, as the first dose capable of decreasing lung endothelial cell viability *in vitro* after 24 h of NE exposure (data not shown). This concentration is in the ng/mL order of magnitude, which is found in critically ill patients [6–8].

### 2.3. Measurement of Reactive Oxygen Species

#### 2.3.1. CellROX**®**

ROS production was assessed by fluorescence via measurement of CellROX® oxidation in a 96-well plate assay according to the manufacturer's instructions. RLMEC (10^3^ cells/well) were serum deprived for 24 hours and stimulated with NE 5 ng/mL for 5 to 120 minutes. To assess if NE induces superoxide anion or hydrogen peroxide generation, RLMEC were preincubated with PEG-SOD (25 U/mL) or PEG-CAT (200 U/mL) for 30 minutes before RLMEC exposure to NE for 5 minutes. Concentrations of PEG-SOD and PEG-CAT were chosen based on the literature [9]. To assess the contribution of *α*- and *β*-adrenoceptors, RLMEC were incubated with phentolamine (10^−8^ mol/L) or propranolol (10^−8^ mol/L) for 30 minutes before exposure to NE for 5 minutes. The concentration on phentolamine and propranolol was chosen based on a concentration-response curve as the lowest dose capable of inhibiting NE-inducing ROS production in RLMEC.

#### 2.3.2. DCFDA

ROS production was assessed by the detection of carboxy-2′,7, dichlorofluorescein fluorescence (DCF) by epifluorescence microscopy. RLMEC were plated in glass coverslips pretreated with 0.4% gelatin. Cells were serum deprived for 24 hours and stimulated with NE 5 ng/mL for 15 minutes. To assess if NE induces superoxide anion or hydrogen peroxide generation, RLMEC were preincubated with PEG-SOD (25 U/mL) or PEG-CAT (200 U/mL) for 30 minutes before RLMEC exposure to NE for 15 minutes. Concentrations of PEG-SOD and PEG-CAT were chosen based on the literature [9]. After stimulation with NE, cells were washed with HBSS buffer and incubated with 25 *μ*M of carboxy-5-(and-6)-carboxy-2′,7′-dichlorodihydrofluorescein (carboxy-DCFDA) for 30 minutes. Cells were fixed in 10% methanol for 5 minutes and mounted in prolong antifade mounting media. Images were acquired by Leica microscope (DMI6000) using LAS X® software. Images were quantified with ImageJ (NIH) software. Data is presented as relative fluorescent units (RFU, fluorescence on 488 channel normalized by the number of cells in the field (assessed by DAPI staining)).

#### 2.3.3. Nox Activity

Nox activity was assessed in membrane fractions as previously described [10, 11]. RLMEC were stimulated with NE (5 ng/mL) for 5 up to 120 minutes. In specific studies, RLMEC were preincubated with phentolamine (10^−8^ mol/L) or propranolol (10^−8^ mol/L) before exposure to NE (5 ng/mL) for 5 minutes. Membrane fractions were isolated from RLMEC using Mem-PER™ Plus® Membrane Protein Extraction Kit according to the manufactures' instructions. Nox activity in membrane fractions was assessed by the kinetics of DHE oxidation in a 96-well plate fluorimeter. One hundred microliters of the membrane extract was added to each well, along with 0.12 *μ*L DHE (10 mM), 25 mg/mL DNA, and 101.88 *μ*L phosphate buffer (50 mM, pH 7.4). Fluorescence was read for 30 min (*λ*_exc_ = 485 nm, *λ*_em_ = 595 nm). Three microliters of NADPH (2 mmol/L) was then added and a new reading was performed for 30 min. The delta for the area under the curve (*Δ*AUC) was calculated (ΔAUC = AUC after NADPH−AUC before NADPH). *Δ*AUC was normalized by protein content in the sample. The results are expressed as percentage of control.

### 2.4. Immunohistochemistry

RLMEC were plated in glass coverslips pretreated with 0.4% gelatin. When cells reached confluence, they were serum deprived for 24 h and incubated with PBS or NE (5 ng/mL) for 15 minutes. Cells were fixed with 4% paraformaldehyde and permeabilized with triton 0.1% in HBSS. Blocking of nonspecific binding sites was done with 5% normal goat serum. Cells were incubated with phospho-p47phox (Ser370) antibody overnight at 4°C. Cells were washed and incubated with Alexa Fluor ™ 647 goat anti-rabbit antibody for 2 hours at room temperature followed up by incubation with DAPI. Slides were mounted and imaged on a Leica microscope (DMI6000) using LAS X® software. Images were quantified with ImageJ (NIH). Data is presented as relative fluorescent units (RFU, fluorescence on 647 channel normalized by the number of cells in the field (assessed by DAPI staining)).

### 2.5. Real-Time PCR

RNA was extracted by TRIZOL® (Invitrogen) and quantified using NanoDrop (Thermo Fisher Scientific). One-step real-time PCR was performed using Power SYBR™ Green RNA to C_T_™ RT-PCR kit (Applied Biosystems by Thermo Fisher Scientific) and the QuantStudio 3 (Thermo Fisher Scientific). Primers were obtained from Integrated DNA Technologies (Coralville, IA). Data were analyzed using the comparative cycle threshold (CT) method. Relative quantification to the control sample calibrator was calculated using the formula 2^(-*ΔΔ*CT)^. Gene expression was normalized to the level of beta actin, which was used as an internal control. Data are presented as relative expression levels compared with the control.

### 2.6. Cell Viability and Cell Death

Cell viability was assessed by trypan blue staining. Results are expressed as percentage of viable cells. Cell death was assessed by annexin V and propidium iodide (PI) staining via flow cytometry using a commercially available kit (FITC Annexin V/Dead Cell Apoptosis Kit®) from Thermo Fisher Scientific. Apoptotic cells were detected by FragEL ™ DNA Fragmentation Detection Kit Fluorescence TdT enzyme according to the manufacturer's instructions and DNA fragmentation was assessed by electrophoresis. For these studies, RLMEC were incubated with NE 5 ng/mL or PBS for 24 hours. When required, cells were preincubated with PEG-SOD (25 U/mL) or PEG-CAT (200 U/mL) for 30 minutes before RLMEC exposure to NE.

### 2.7. Caspase Activation

Caspase-3 and caspase-7 activation were assessed by fluorescence microscopy using CellEvent™ kit (Invitrogen). RLMEC were exposed to NE 5 ng/mL for 24 hours in the presence or absence of phentolamine (10^−8^ mol/L), apocynin (30 *μ*mol/L), PEG-SOD (25 U/mL), or PEG-CAT (200 U/mL). Images were acquired by an upright fluorescence microscope (Olympus BX51) using Olympus Screen Saver software (Olympus).

### 2.8. Statistical Analysis

Data are presented as mean ± SD. Groups were compared using 1-way ANOVA or Student *t* test, as appropriate. Newman-Keuls post hoc test was used to compensate for multiple testing procedures. *p* < 0.05 was considered statistically significant.

## 3. Results

### 3.1. ROS Production

**Figure 1:**
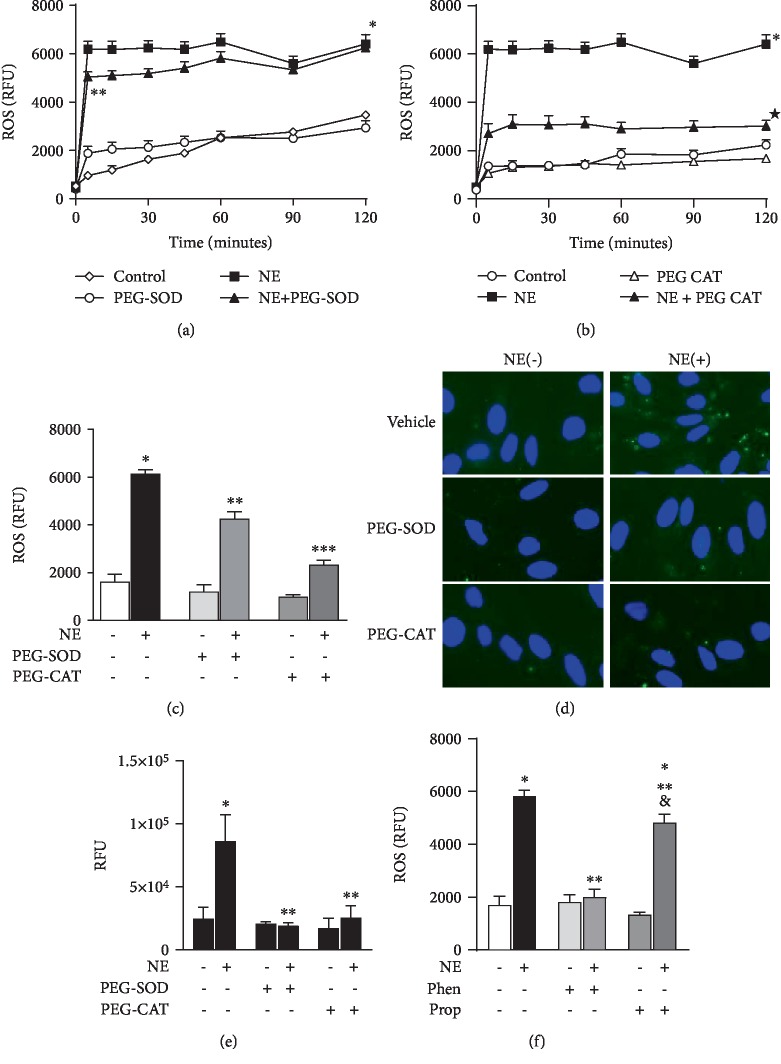
Norepinephrine induces ROS production in rat lung microvascular endothelial cells. Rat lung microvascular endothelial cells (RLMEC) were stimulated with norepinephrine (NE) 5 ng/mL for 120 minutes. (a) NE increases ROS production in RLMEC, an effect inhibited in the presence of PEG-SOD. (b) ROS levels are decreased in the presence of PEG-CAT. (c) Comparative graph illustrating the effects of PEG-SOD and PEG-CAT on ROS production stimulated by the exposure of RLMEC to NE for 5 minutes. (d) Representative images of ROS production by NE in RLMEC assessed by DCF fluorescence; effects of PEG-SOD and PEG-catalase are also shown. (e) Quantification of ROS production assessed by DCF fluorescence: RLMEC were stimulated with NE 5 ng/mL for 15 min in the presence and absence of PEG-SOD and PEG-CAT. (f) Phentolamine (Phen) and propranolol (Prop) had no effects on basal ROS production; Phen inhibited NE-induced ROS production in RLMEC and Prop partly inhibited NE-induced ROS production on RLMEC. ^∗^*p* < 0.05*vs*. control, ^∗∗^*p* < 0.05*vs*. NE, ^∗∗∗^*p* < 0.05*vs*. NE+PEG-SOD, ^&^*p* < 0.05*vs*. propranolol. *N* ≥ 5/group.

ROS production was measured in a real-time manner using CellROX® probe (Thermo Fisher Scientific) in RLMEC stimulated or not with NE. A two-fold increase in ROS production was observed as early as 5 minutes after exposure to NE (3223.8 ± 631.55 RFU in the NE group *vs*. 1072.9 ± 520.29 RFU in the control group); steady ROS production lasted for 2 hours (Figure 1(a)). ROS production was partly inhibited by PEG-SOD (NE + PEG‐SOD = 4257 ± 800.0 RFU *vs*. NE = 6149 ± 626.2 RFU) (Figure 1(a)) and PEG-catalase (NE + PEG‐CAT = 2330 ± 473.4 RFU *vs*. NE = 6149 ± 626.2 RFU) (Figure 1(b)) indicating that NE leads to the generation of both superoxide anion and hydrogen peroxide. Incubation of RLMEC with PEG-SOD or PEG-CAT only did not alter ROS production (PEG‐SOD = 1204 ± 756.4 RFU and PEG‐CAT = 988.7 ± 208.7 RFU) (Figures 1(a)–1(c)). To confirm the effects of NE on ROS production, we assessed ROS generation via a second methodology, oxidation of DCFDA by epifluorescence microscopy. NE increased ROS production (NE = 86350 ± 9380 RFU *vs*. vehicle = 24677 ± 4712 RFU) in RLMEC, an effect inhibited by both PEG-SOD and PEG-CAT (19138 ± 2531 and 25710 ± 9278, respectively) (Figures 1(d) and 1(e)). Next, to determine the adrenoceptor involved, cells were incubated with phentolamine (10^−8^ mol/L) or propranolol (10^−8^ mol/L) for 30 minutes before stimulation with NE for 5 minutes. Incubation of RLMEC with only phentolamine (Phen = 1814 ± 285.4) or propranolol (Prop = 1332 ± 94.8) did not alter ROS levels in RLMEC. Cells were then stimulated with NE+phentolamine or NE+propranolol. Whereas phentolamine fully blocked the effects of NE on ROS production (NE + Phen = 2003 ± 290.5*vs*. NE = 5832 ± 215.0), propranolol partly inhibited ROS production by NE (NE + Prop = 4815 ± 324.3*vs*. NE = 5832 ± 215.0) (Figure 1(f)).

### 3.2. Nox Activity

**Figure 2:**
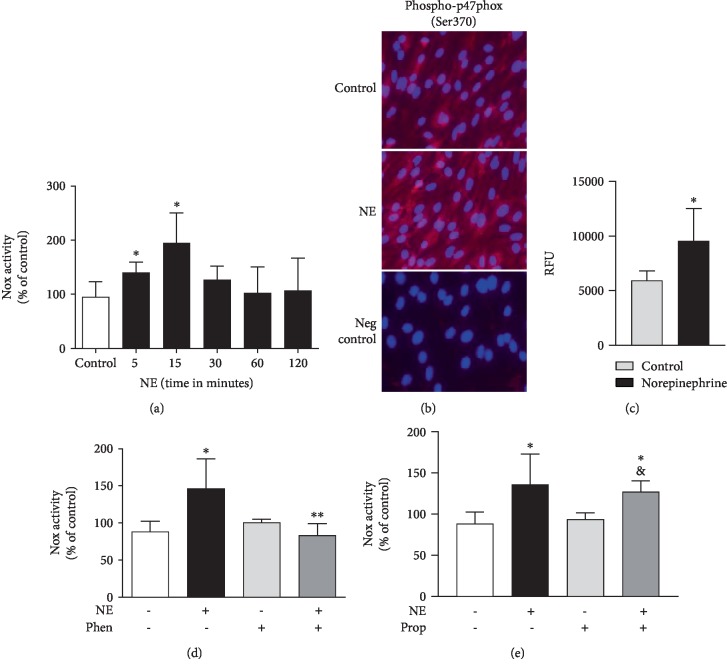
Norepinephrine activates Nox via *α*-adrenergic receptors. Rat lung microvascular endothelial cells (RLMEC) were stimulated with norepinephrine (NE) 5 ng/mL for 5 to 120 minutes. (a) NE increases Nox activity in RLMEC after 5 and 15 minutes of stimulation. (b) Representative images: NE increases p47phox phosphorylation at Ser370 in RLMEC after 15 minutes of incubation. (c) Quantification of p47phox phosphorylation in RLMEC stimulated or not with NE for 15 minutes: RFU (relative fluorescent units) is the ratio of fluorescence intensity/cell number of a given field in cells treated or not with NE. (d) Phentolamine (Phen), an antagonist of *α*_1_-adrenoceptors, inhibits NE-induced Nox activation. (e) Propranolol (Prop), a nonspecific *β*-adrenoceptor antagonist, do not inhibit NE-induced Nox activation. ^∗^*p* < 0.05*vs*. control; ^∗∗^*p* < 0.05*vs*. NE; ^&^*p* < 0.05*vs*. prop. *N* ≥ 5/group.

NE increased Nox activity in isolated membrane fractions after 5 minutes (in % of control: NE = 140.6 ± 19.12); activity peaked at 15 minutes (NE = 195.4 ± 54.62%) and decreased after 30 minutes (NE = 126.6 ± 25.26%) (Figure 2(a)). Additionally, exposure of RLMEC to NE for 15 minutes increased phosphorylation of p47phox at Ser370 (Figures 2(b) and 2(c)). To assess the contribution of alpha-adrenergic receptor, Nox activity was measured in the presence of phentolamine. Phentolamine completely blocked the increase in Nox activity after exposure to NE (NE + Phen = 83.2 ± 16.01% *vs*. NE = 146.3 ± 40.13%) (Figure 2(d)). To evaluate the role of *β*-adrenoceptors in NE-induced Nox activation, cells were treated with propranolol, a nonselective *β*-adrenoceptor antagonist. Propranolol did not inhibit the effects of NE on Nox activity (in %: NE + Prop = 127.3 ± 13.03*vs*. *NE* = 136.3 ± 36.96) (Figure 2(e)). Incubation of RLMEC with phentolamine or propranolol only did not alter Nox activity (Phen = 100.8 ± 4.28% and Prop = 93.84 ± 7.922%) (Figures 2(d) and 2(e), respectively).

### 3.3. Nox Expression

**Figure 3:**
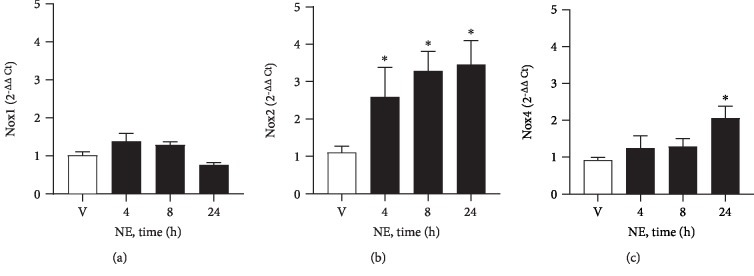
NE increases Nox2 and Nox4 mRNA expression. Rat lung microvascular endothelial cells (RLMEC) were stimulated with norepinephrine (NE, 5 ng/mL) or vehicle (V) for 4 to 24 hours (h). (a) NE does not alter mRNA expression of Nox1. (b) NE increases mRNA expression of Nox2 after 24 hours. (c) NE increased mRNA expression of Nox4 in a time-dependent manner. ^∗^*p* < 0.05 vs. vehicle (V). *N* = 5/group.

Incubation of RLMEC with NE increased Nox2 and Nox4 mRNA expression in RLMEC in a time-dependent manner. Nox2 was the first isoform to show increased content after 4 hours (2.5 delta increase relative to control) of RLMEC incubation with NE. Importantly, a gradual increase pattern in Nox2 mRNA was observed (3.29-fold at 8 h and 3.5-fold at 24 h). Nox4 was augmented only 24 hours after cells exposure to NE (2.0-fold). Nox1 mRNA expression was not altered by NE (Figures 3(a)–3(c)).

### 3.4. NE Induces RLMEC Death

**Figure 4:**
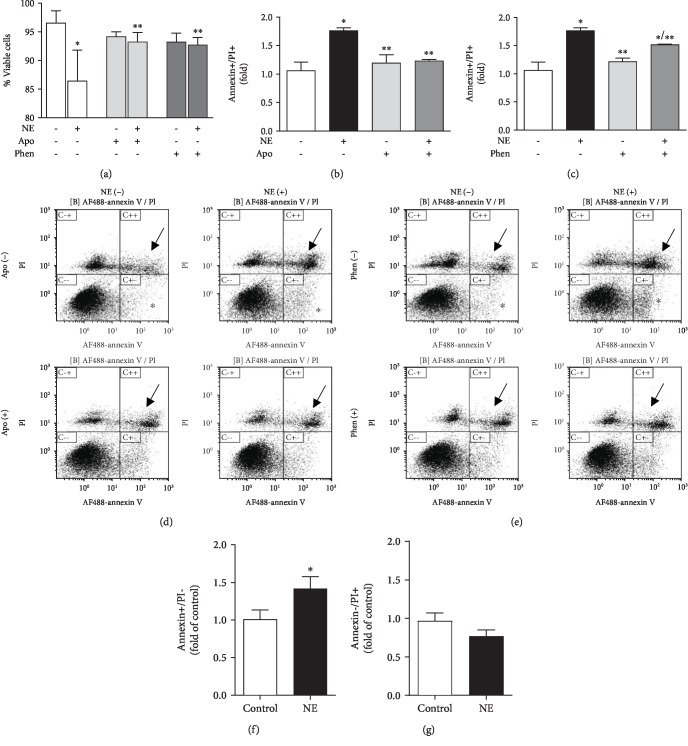
Norepinephrine induces RLMEC death via ROS production and *α*_1_-adrenoceptor-mediated pathways. To assess the role of reactive oxygen species and *α*_1_-adrenoceptors in NE-induced cells death, RLMEC were treated with apocynin (Apo) and phentolamine (Phen) for 30 minutes before stimulation with NE (5 ng/mL) for 24 h. (a) NE reduced cell viability as assessed by trypan blue, an effect inhibited by Apo and Phen. (b). NE induces cell death as it increases the amount of annexin+/PI+ cells, an effect is abolished by Apo. (c). Phen partly inhibits NE-induced cell death. (d, e) Representative flow cytometry charts evidencing that NE increases the number of annexin+/PI+ cells (arrows) and the number of annexin-positive cells (stars); apocynin reduces the number of annexin+/PI+ cells to control levels, and Phen decreased the number of annexin+/PI+cells in 25%. (f) Bar graph indicating that NE increases the number of annexin+/PI- cells. (g) Bar graph indicating that NE does not alter the number of annexin-/PI+ cells. ^∗^*p* < 0.05*vs*. control, ^∗∗^*p* < 0.05*vs*. NE; *N* ≥ 4/group.

NE decreased the number of viable cells by 10% when compared to control (NE = 86.4 ± 5.41% *vs*. control = 96.5 ± 2.13%) as assessed by trypan blue. In the presence of apocynin (30 *μ*mol/L), a ROS scavenger in vascular cells, the deleterious effects of NE on cell viability were abolished (NE + APO = 93.3 ± 1.6% *vs*. NE = 86.4 ± 5.41%). Phentolamine inhibited the deleterious effects of NE on RLMEC viability (*Phen* + NE = 92.7 ± 1.26% vs. NE = 86.4 ± 5.41%). Apocynin (Apo = 94.2 ± 0.84% *vs*. control = 96.5 ± 2.13%) or phentolamine (Phen = 93.2 ± 1.5% *vs*. control = 96.5 ± 2.13%) alone had no effects on cell viability (Figure 4(a)).

To further investigate the effects of NE on cell death, we assessed staining of RLMEC with propidium iodide and annexin V by flow cytometry. NE increased the number of annexin+/PI+ cells by 70% when compared to control (NE = 1.8 ± 0.05*vs*. control = 1.1 ± 0.14). In the presence of apocynin, NE-induced cell death, as measured by the number of annexin+/PI+ cells, was inhibited by 50% (NE + Apo = 1.2 ± 0.02*vs*. NE = 1.8 ± 0.05). Apocynin alone had no effect on RLMEC cell death (Apo = 1.1 ± 0.14*vs*. control = 1.1 ± 0.14) (Figures 4(b) and 4(d)).

To confirm that NE-induced cell death was mediated by alpha-adrenergic receptor activation, we measured cell death in the presence of phentolamine. Phentolamine inhibited NE-induced cell death by approximately 25% (NE + Phen = 1.51 ± 0.01*vs.*NE = 1.77 ± 0.05). Phentolamine alone had no effect on RLMEC death (Phen = 1.21 ± 0.07) (Figures 4(c) and 4(e)).

NE also increased the number of annexin+/PI- cells by 40% compared to control cells (NE = 1.4 ± 0.16*vs*. control = 1.0 ± 0.18). This effect was not inhibited by either apocynin (NE + Apo = 1.3 ± 0.1) or phentolamine (NE + Phen = 1.7 ± 0.2) (Figure 4(f)). NE did not alter the number of annexin-/PI+ cells (NE = 0.8 ± 0.08*vs*. control = 0.9 ± 0.10) (Figure 4(g)).

**Figure 5:**
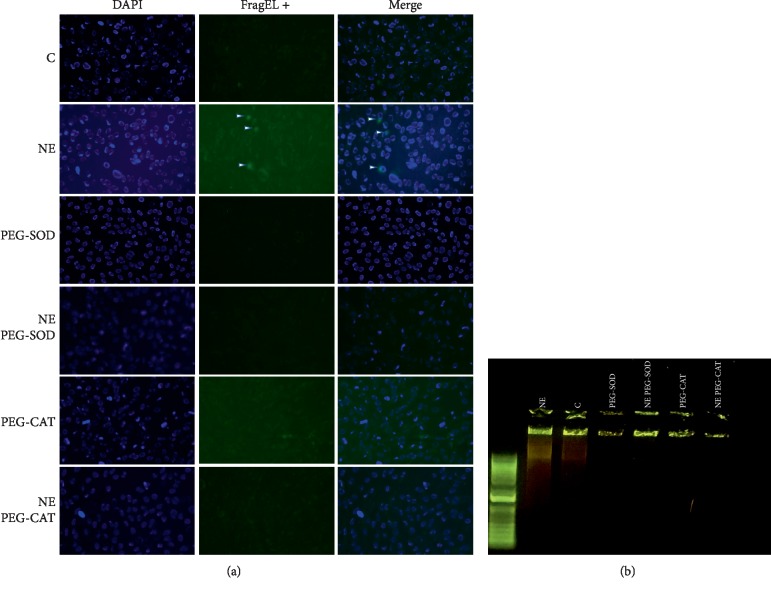
Superoxide anion and hydrogen peroxide are involved in NE-induced RLMEC death. RLMEC were stimulated with NE (5 ng/mL) for 24 h. Detection of apoptotic cells was performed using (a) FragEL kit (Millipore) and by (b) DNA fragmentation. NE induced the appearance of RLMEC in late apoptotic stages as evidenced by both the FragEL and DNA fragmentation assays. These effects were inhibited in the presence pf PEG-SOD and PEG-CAT.

In order to pinpoint which Nox-derived reactive oxygen species mediate NE deleterious effects on RLMEC, we assessed RLMEC death in the presence of PEG-SOD and PEG-CAT by two different methodologies: the detection of apoptotic cells by the FragEL DNA fragmentation commercial kit and assessment of DNA fragmentation via electrophoresis. NE induced both DNA fragmentation and appearance of cells in the late apoptotic stage. Those effects were inhibited by PEG-SOD and PEG-CAT (Figures 5(a) and 5(b)).

### 3.5. Norepinephrine Regulates Caspase-3 by ROS and Alpha-Adrenoceptor-Dependent Mechanisms

**Figure 6:**
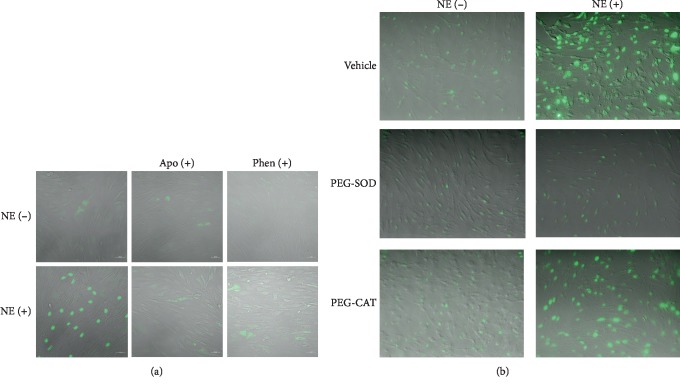
Norepinephrine activates caspase-3 via ROS production and *α*_1_-adrenoceptor-mediated pathways. To assess the role of reactive oxygen species and *α*_1_-adrenoceptors in norepinephrine- (NE-) induced caspase-3 activation, RLMEC were treated with apocynin (Apo), phentolamine (Phen), PEG-SOD, or PEG-CAT for 30 minutes before stimulation with NE (5 ng/mL) for 24 h. NE induces caspase-3 translocation to the nucleus, an effect inhibited by (a) Apo and Phen and (b) PEG-SOD. PEG-CAT did not inhibit caspase-3 activation by NE. *N* = 3/group.

To assess the role of reactive oxygen species and *α*_1_-adrenoceptors in norepinephrine (NE)-induced caspase-3 activation, RLMEC were treated with apocynin (Apo), phentolamine (Phen), PEG-SOD, or PEG-CAT for 30 minutes before stimulation with NE (5 ng/mL) for 24 h. NE induced the translocation of caspase-3 to the nucleus, an index of caspase-3 activity (Figure 6). Incubation with either apocynin or phentolamine (Figure 6(a)) inhibited nuclear staining of caspase-3. In the same manner, incubation of RLMEC with PEG-SOD inhibited NE-induced caspase-3 activation. Incubation of RLMEC with PEG-CAT had no effect on caspase-3 activation by NE (Figure 6(b)).

### 3.6. Norepinephrine Induces Rat Lung Microvascular Endothelial Cell Death by Caspase-3-Dependent Mechanisms

**Figure 7:**
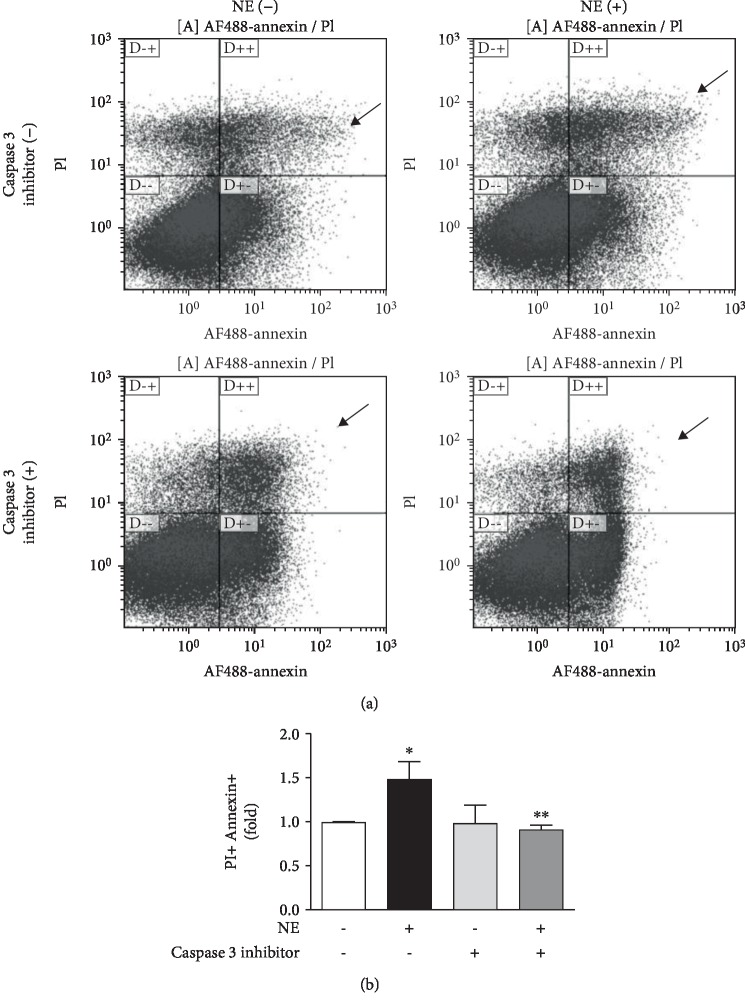
Norepinephrine induces cell death via caspase-3-dependent pathways. Rat lung microvascular endothelial cell (RLMEC) death was assessed by measurement of annexin and propidium iodide staining by flow cytometry after stimulation with norepinephrine (5 ng/mL). To assess the role of caspase-3 in NE-induced cell death, RLMEC were treated with caspase-3 inhibitor. (a) NE increases the number of annexin+/PI+ cells as indicates by arrows. (b) Bar graph corresponding to the quantification of annexin+/PI+ cells in cells treated and nontreated with NE, in the presence or absence of caspase-3 inhibitor. ^∗^*p* < 0.05*vs*. control and ^∗∗^*p* < 0.05*vs*. NE; *N* ≥ 3/group.

NE increased the number of annexin+/PI+ cells identified via flow cytometry when compared to untreated cells (NE = 1.48 ± 0.19*vs*. control = 0.99 ± 0.01). In the presence of caspase-3 inhibitor, NE-induced cell death returned to control levels (NE + Casp 3 Inhibitor = 0.92 ± 0.04). Caspase-3 inhibitor had no effects *per se* on the number of annexin+/PI+ cells (Casp 3 inhibitor = 0.98 ± 0.21*vs*. control = 0.99 ± 0.01). No changes were detected in the number of annexin+/PI- or annexin-/PI+ cells (Figures 7(a) and 7(b)).

## 4. Discussion

The major finding of this study was that NE, at a concentration in the order of magnitude found in plasma of critically ill patients, has deleterious effects on lung microvascular endothelial cells. NE is routinely used in the clinical settings to treat hypotension, at significant higher concentrations, and the immediate effects of NE appear beneficial insofar as NE improves blood pressure and, possibly, short-term patient survival. For example, studies in rats indicate that typical pressor doses of NE can alter pulmonary capillary integrity leading to accumulation of lung water and ultimately pulmonary edema [1, 2]. We therefore investigated the deleterious effects of a clinically relevant concentration of NE on lung endothelial cells. Our findings demonstrate that NE triggers Nox activation and ROS production via *α*_1_-adrenoceptors and that the onset of oxidative stress leads to caspase-3 activation, mainly by superoxide anion, which ultimately causes RLMEC death. Collectively, these data suggest that concentrations of NE typically found in critically ill patients may be deleterious to lung endothelial cells.

NE is known to produce ROS in a broad range of cell types via the activation of different adrenoceptors. Deo and collaborators showed that NE increases ROS production in human peripheral blood mononuclear cells (PBMCs) via *α*_2_-adrenoceptors [3]. NE also induces ROS generation in U937 macrophages via beta-adrenergic signaling as evidenced by the reversal of ROS production to basal levels when U937 cells were pretreated with propranolol [5]. Here, we show that, in RLMEC, NE induces Nox-dependent ROS production by activation of *α*_1_-adrenoceptors since phentolamine, a specific *α*_1_-adrenoceptor antagonist, abolishes NE-induced ROS production. Furthermore, incubation of RLMEC with propranolol, a nonspecific antagonist of *β*-adrenoceptors, did not inhibit NE- induced Nox activation. In addition, we found that incubation of RLMEC with either PEG-SOD or PEG-catalase partly inhibits NE-induced ROS production indicating NE can promote generation of both superoxide anion and hydrogen peroxide. Propranolol, a nonselective *β*-adrenoceptor antagonist, partly inhibited ROS production by NE but did not alter the increase in Nox activity induced by NE, suggesting that exposure to NE may result in increased ROS production by different pathways in RLMEC. Herein, we focused on investigating how Nox-driven ROS may contribute to cell death in RLMEC exposed to NE.

Nox is the main source of ROS in the vasculature. Interestingly, different Nox isoforms preferentially generate hydrogen peroxide over superoxide anion. Nox2 is the main isoform present in endothelial cells and it is known to constitutively generate superoxide anion in basal conditions. The fact that we detected increased production of both superoxide anion and hydrogen peroxide in cells treated with NE suggests that a different source of ROS, such as mitochondrial, may also participate in the deleterious effects of NE. Our findings show that apocynin, a ROS scavenger/antioxidant in vascular cells, decreases the number of annexin+/PI+ cells but does not change the number of annexin+/PI- cells. Moreover, incubation of RLMEC with either PEG-SOD or PEG-CAT inhibit the effects of NE on DNA fragmentation. These findings support the idea that NE may act via additional pathways that contribute to its toxic effects on endothelial cells. It is important to mention that the effects of apocynin in vascular cells is not directly related to Nox activity as vascular cells lack myeloperoxidase and are not able to dimerize apocynin [12]. Further studies are required to identify additional mechanisms of NE actions on endothelial cells. NE has been shown to increase ROS generation in cardiac endothelial cells, albeit at concentrations over 1000-fold higher than we used in this study [13]. Xiong and collaborators showed that NE augments ROS production in embryonic ventricular myocyte H9c2 cells via selective increase in Nox1 expression [4], but others found that NE-induced ROS production occurs by Nox2-dependent pathways in adult cardiac myocytes [14, 15]. In PBMCs, ROS production was associated with increased gp91phox, p22phox, and p67phox mRNA expressions. Collectively, these reports corroborate our findings that NE induces ROS production via Nox-dependent mechanisms. Notably, ours is the first report to highlight the effects of physiological concentrations of NE on ROS production and Nox activity.

The effects of ROS on the vasculature vary according to source and cell type. Herein, we show that NE-induced ROS results in caspase-3 activation and RLMEC death. Our data corroborate studies in the literature that report ROS activators of caspase-3-mediated apoptosis is triggered by different stimuli. Particularly in EC, ROS have been shown to mediate TNF-induced Nox4 caspase-3 activity and Bcl1 expression in HUVEC [16]. Tian and collaborators also reported a role for Nox4 in caspase-mediated endothelial cell death in inflammatory conditions [17]. Park and collaborators reported the specific role of superoxide anion in lysophosphatidylcholine-induced caspase-3 activation and apoptosis in HUVEC [18]. Likewise, Tawfik and colleagues reported that exogenous addition of hydrogen peroxide or hyperglycemia-stimulated ROS activates caspase3-induced cell death via JAK2-dependent pathways in aortic endothelial cells [19]. Our data indicate that NE induces production of both superoxide anion and hydrogen peroxide via Nox2- and Nox4-dependent mechanisms.

Fu and collaborators reported that micromolar concentrations of NE induce neonatal rat endothelial cell death by ROS-dependent activation of c-jun N-terminal kinase (JNK) [13]. The same group also showed that this effect is mediated by caspases 2 and 3 via beta-adrenergic signaling, with no contribution of *α*-adrenoceptors [20, 21]. Taken together, these data support our findings of NE increasing ROS production by alpha- and beta-adrenoceptor signaling and corroborate our finding that in NE-induced caspase-3 activation. They have also reported that production of ROS by NE is a NADPH-independent mechanism. The fact that they have not found a role for alpha-adrenoceptor signaling in NE-induced apoptosis and described a different pathway for ROS formation can be potentially related to the adrenoceptor subtype in the studied cell type and or be a tissue-dependent phenomenon.

Herein, we showed that NE can result in RLMEC death by two different assessments, cell viability via trypan blue staining and cell death by annexin V and PI double positive staining. Annexin V is an early marker for apoptosis but may occur in late stages of different forms of cell death such as necrosis. In the same way, PI positive staining is usually a marker for necrosis but it may occur in the later stages of apoptosis. Further studies are necessary to clarify what type of cell death is predominant when endothelial cells are exposed to clinically found concentrations of NE.

## 5. Conclusion and Clinical Significance

The results presented here demonstrate a potential adverse effect of NE at concentrations expected in a clinical setting. These findings suggest that exposure of lung endothelial cells to supraphysiological doses of NE could be harmful to patients as it triggers lung endothelial cells death. Further studies are necessary to address the potential deleterious effects of NE in clinical settings.

## Data Availability

The data used to support the findings of this study are available from the corresponding author upon request

## Abbreviations

PEG-catalase: Catalase-polyethylene glycol
DHE: Dihydroethidium
NADPH: Dihydronicotinamide-adenine dinucleotide phosphate
RLMEC: Rat lung microvascular endothelial cells
Nox: NADPH oxidase
NE: Norepinephrine
PI: Propidium iodide
ROS: Reactive oxygen species
RIPA buffer: Radioimmunoprecipitation assay buffer
PEG-SOD: Superoxide dismutase-polyethylene glycol
TBS-T: Tris-buffered saline-Tween 20.
